# PREPARING LEADERS IN GERIATRICS HEALTH CARE THROUGH THE VA’S ADVANCED FELLOWSHIP IN GERIATRICS

**DOI:** 10.1093/geroni/igae098.0903

**Published:** 2024-12-31

**Authors:** Chelsea Hawley, Michelle Martinchek, Colleen Hursh, Jennifer Moye, Jennifer Pruskowski, Benjamin Congedo, Shawn Dunlap, Robin Jump

**Affiliations:** National Coordinating Center for the Advanced Fellowship in Geriatrics, Bedford, Massachusetts, United States; VA Boston Healthcare System, Boston, Massachusetts, United States; VA Bedford Healthcare System, Bedford, Massachusetts, United States; VA Boston Healthcare System, Boston, Massachusetts, United States; University of Pittsburgh, Pittsburgh, Pennsylvania, United States; VA Pittsburgh Healthcare System, Pittsburgh, Pennsylvania, United States; VA Bedford Healthcare System, Bedford, Massachusetts, United States; VA Pittsburgh Healthcare System, Pittsburgh, Pennsylvania, United States

## Abstract

The VA Advanced Fellowship in Geriatrics (AFiG) prepares leaders in geriatric research, education, and clinical innovation, to meet the complex needs of the rapidly growing older Veteran population. This interprofessional, two-year fellowship welcomes physicians, nurses, psychologists, and other associated health fellows, including but not limited to pharmacists, social workers, physical and occupational therapists, and non-clinician scientists. AFiG is funded by VA’s OAA, and fellows are hosted by the 20 GRECCs, which are VA Centers of Excellence focused on aging. Fellows devote 75-80 percent of time to scholarly pursuits and 20-25 percent to clinical work (or activities supporting clinical care for non-clinician scientists), working closely with mentors to develop an individualized training experience. AFiG began in 2001 with seven sites and has grown to 20 sites as of 2024. In 2022, a new national Coordinating Center (CC) for AFiG was established to support and empower all fellowship sites to align their training with the fellowship mission and strengths of their individual sites. This presentation will describe efforts to foster a community of practice and increased collaboration, including national curriculum development, facilitation of networking and professional events, and curation of educational opportunities. The CC is also prioritizing a new national recruitment initiative to increase the number and diversity of applicants and to facilitate hiring fellowship graduates into VA positions, which will be discussed. With these such efforts, the CC is further strengthening AFiG as a nationally recognized leader in training future leaders in geriatric healthcare.

